# A draft phased assembly of the diploid Cascade hop (*Humulus lupulus*) genome

**DOI:** 10.1002/tpg2.20072

**Published:** 2021-02-18

**Authors:** Lillian K. Padgitt‐Cobb, Sarah B. Kingan, Jackson Wells, Justin Elser, Brent Kronmiller, Daniel Moore, Gregory Concepcion, Paul Peluso, David Rank, Pankaj Jaiswal, John Henning, David A. Hendrix

**Affiliations:** ^1^ Department of Biochemistry and Biophysics Oregon State University Corvallis OR 97331 USA; ^2^ Pacific Biosciences of California Menlo Park CA 94025 USA; ^3^ Center for Genome Research and Biocomputing Oregon State University Corvallis OR 97331 USA; ^4^ Department of Botany and Plant Pathology Oregon State University Corvallis OR 97331 USA; ^5^ USDA‐ARS‐FSCR Corvallis OR 97331 USA; ^6^ School of Electrical Engineering and Computer Science Oregon State University Corvallis OR 97331 USA

## Abstract

Hop (*Humulus lupulus* L. var Lupulus) is a diploid, dioecious plant with a history of cultivation spanning more than one thousand years. Hop cones are valued for their use in brewing and contain compounds of therapeutic interest including xanthohumol. Efforts to determine how biochemical pathways responsible for desirable traits are regulated have been challenged by the large (2.8 Gb), repetitive, and heterozygous genome of hop. We present a draft haplotype‐phased assembly of the Cascade cultivar genome. Our draft assembly and annotation of the Cascade genome is the most extensive representation of the hop genome to date. PacBio long‐read sequences from hop were assembled with FALCON and partially phased with FALCON‐Unzip. Comparative analysis of haplotype sequences provides insight into selective pressures that have driven evolution in hop. We discovered genes with greater sequence divergence enriched for stress‐response, growth, and flowering functions in the draft phased assembly. With improved resolution of long terminal retrotransposons (LTRs) due to long‐read sequencing, we found that hop is over 70% repetitive. We identified a homolog of cannabidiolic acid synthase (CBDAS) that is expressed in multiple tissues. The approaches we developed to analyze the draft phased assembly serve to deepen our understanding of the genomic landscape of hop and may have broader applicability to the study of other large, complex genomes.

## Core Ideas

- We present an improved reference genome assembly of the hop that better characterizes the genome heterozygosity and gene and repeat content.
- We estimate that the repeat content of the hop genome is over 70%.
- Genes with high divergence between haplotypes are enriched for growth and defense functions.
- We found a homolog of cannabidiolic acid synthase that is expressed in multiple tissues.
- We developed broadly applicable approaches to analyze phased genome assemblies.

**Abbreviations**
BBE‐like: berberine bridge enzyme‐like
BUSCO: benchmarking universal single‐copy orthologs
CBDAS: cannabidiolic acid synthase
HPC: homologous primary contig
LTR: long terminal retrotransposon
SMRT sequencing: Single molecule real‐time
SNP: single nucleotide polymorphism
SV: structural variant
TE: transposable element
WGD: whole genome duplication.

## 1 INTRODUCTION

Hop (*Humulus lupulus* L. var Lupulus) is a diploid (2n = 18+XX/XY) (Grabowska‐Joachimiak, Sliwinska, Pigula, Skomra, & Joachimiak, 2006), dioecious plant with a history of cultivation spanning over one thousand years (Behre, 1999; Wilson, 1975; Zanoli & Zavatti, 2008). Large size (2.8 Gb) (Zonneveld, Leitch, & Bennett, 2005), abundant repeat content, and heterozygosity have challenged draft assemblies of the hop genome (Hill, Sudarsanam, Henning, & Hendrix, 2017; Natsume et al., 2014). Hop cones from female plants, commonly called “hops,” are valued for their use in brewing, and hop has been used in traditional medicine to treat a variety of ailments, including anxiety, insomnia, and pain. Recent in vitro and animal model studies have revealed compounds of medicinal interest in hop that exhibit activity against metabolic syndrome (Miranda et al., 2018; Van Cleemput et al., 2009; Yajima et al., 2004), cancer (Jiang, Sun, Xiang, Wei, & Li, 2018; Miranda et al., 1999; Zanoli & Zavatti, 2008), microbes (Haas & Barsoumian, 1994; Van Cleemput et al., 2009; Zanoli & Zavatti, 2008), and possess estrogenic (Chadwick, Pauli, & Farnsworth, 2006; Milligan et al., 2000; Possemiers et al., 2006; Stevens & Page, 2004) properties. Cascade is a backcross hybrid of English and Russian varieties of hop (Lemmens, 1998) and is the most widely used hop cultivar in craft brewing (Gent, Massie, Twomey, & Wolfenbarger, 2017).

Hop cones are rich in secondary metabolites, including bitter acids, terpenes, and polyphenols (De Keukeleire et al., 2003; Kavalier et al., 2011; Van Cleemput et al., 2009). Hop was originally added to beer for its preservative activity and flavor, due to the presence of bitter acids, including alpha and beta acids, found in hop cones. Alpha acids, including humulone, cohumulone, and adhumulone, exhibit antimicrobial activity and contribute to beer foam stability (Hieronymus, 2012; Verzele & De Keukeleire, 2013; Zanoli & Zavatti, 2008). Isomerization of alpha acids to iso‐alpha acids at high pH and temperature results in a characteristic bitter flavor (Zanoli & Zavatti, 2008). The most abundant terpenes are monoterpene beta‐myrcene, as well as sesquiterpenes alpha‐humulene and beta‐caryophyllene (Almaguer, Schönberger, Gastl, Arendt, & Becker, 2014). Hop also contains a variety of flavonoids (Dostálek, Karabín, & Jelínek, 2017), which are known to have healthful properties (Bondonno et al., 2019). The most abundant prenylflavonoid in hop is xanthohumol (XN), which has shown anti‐cancer activity in animal model and in vitro studies (Jiang et al., 2018). Hop produces 8‐prenylnaringenin (8‐PN), which has strong estrogenic activity and potential for pharmacological applications (Štulíková, Karabín, Nešpor, & Dostálek, 2018). An improved assembly will inform the development of new cultivars optimized to yield desired metabolites, and will help to identify regulatory regions that modulate transcription of genes involved in the biosynthesis of these compounds.

Powdery mildew reduces the quality and quantity of yield while increasing the cost of production (Henning et al., 2017a). Although Cascade has possessed disease resistance to fungal pathogens powdery mildew (*Podosphaera macularis*) (Gent et al., 2017) and downy mildew (*Pseudoperonospora humuli*) (Henning et al., 2015), these fungal pathogens have the potential to evolve and acquire resistance. A recent outbreak of powdery mildew affecting Cascade in the U.S. Pacific Northwest (PNW) was the result of a Cascade‐adapted isolate of *P. macularis* (Gent et al., 2017). Very little management was required to grow Cascade until powdery mildew was introduced to the PNW in the mid‐1990s (Ocamb, Klein, Barbour, Griesbach, & Mahaffee, 1999), and the rise of PM infection corresponds to an increase in Cascade acreage (Gent et al., 2017). Hop breeding with resistant cultivars (Henning et al., 2017a; Henning et al., 2015; Neve, 1991) may result in new resistance, but given the inevitability of pathogen adaptation, an understanding of the genomic features underlying resistance and susceptibility is necessary to breed for hop that can withstand pathogen adaptations. Past efforts to identify alleles, genes, and regulatory regions have been hindered by incomplete draft assemblies (Henning et al., 2017a). Insufficient genome coverage was cited as the reason for difficulty detecting an R‐gene quantitative trait locus (QTL) for powdery mildew in hop (Henning et al., 2011). To improve strategies for breeding disease‐resistant hop, and to lay the foundation for future work toward identifying QTLs associated with disease susceptibility and resistance, we sought to create an updated, more‐complete assembly.

**Core Ideas**

- We present an improved reference genome assembly of the hop that better characterizes the genome heterozygosity and gene and repeat content.
- We estimate that the repeat content of the hop genome is over 70%.
- Genes with high divergence between haplotypes are enriched for growth and defense functions.
- We found a homolog of cannabidiolic acid synthase that is expressed in multiple tissues.
- We developed broadly applicable approaches to analyze phased genome assemblies.

Recent analyses of high‐quality assemblies of cultivated plant species have revealed that genes and regulatory elements associated with desired traits are often located near long terminal retrotransposons (LTRs) (Butelli et al., 2012; Grassa et al., 2018; Kobayashi, Goto‐Yamamoto, & Hirochika, 2004; Laverty et al., 2019; Zhang et al., 2019). LTRs are the most abundant type of transposable element (TE) in plant genomes and are largely responsible for the expansion of genome size, as they duplicate via a copy‐and‐paste mechanism (Sanmiguel & Bennetzen, 1998). Proliferation of TEs can occur as a response to biotic and abiotic stresses (Vicient & Casacuberta, 2017), and TE abundance in large plant genomes is often more than 80% (Oliver, McComb, & Greene, 2013). TE proliferation is associated with a variety of changes to the genome, including gene regulation, duplication, rearrangement, and interruption of gene function (Rebollo, Romanish, & Mager, 2012). In plant genomes, the majority of genes are duplicated (Guo, 2013). Most duplicated genes in plants are derived from whole genome duplication (WGD) events, transposon movement, and unequal crossing‐over (Panchy, Lehti‐Shiu, & Shiu, 2016). Duplicated genes provide a mechanism for plants to adapt to biotic and abiotic stresses, and are associated with speciation and diversity among plant species (Panchy et al., 2016).

Most assemblies of diploid species are collapsed, haploid representations of the genome, consisting of merged consensus sequences that represent neither haplotype fully. Collapsed assemblies lose haplotype‐specific information, confounding the identification of single nucleotide polymorphisms (SNPs) and structural variants. Regions of heterozygosity can also break contigs, introducing additional complexity into the assembly, and are particularly detrimental for assembling a large and highly heterozygous genome such as the hop genome. Previous attempts at assembling the *Humulus lupulus* var. lupulus cv. Shinshuwase (hereafter referred to as Shinshuwase) and Teamaker cultivars of the hop genome with short reads have been fragmented and incomplete, containing collapsed regions that thwart the potential investigation of repeats, genes embedded within repeats, gene copy number, and heterozygosity (Hill et al., 2017; Natsume et al., 2014).

Previously, the hop genome was estimated to contain ∼60% repeat sequences, based on a clustering analysis of genome short sequencing reads from *Humulus lupulus* var. cordifolius and Shinshuwase. Similarity to repeat sequences was identified using RepBase and Conserved Domain Database (Pisupati, Vergara, & Kane, 2018). In the Shinshuwase short‐read assembly, 34.7% of total scaffold length was composed of repeat sequences, with 93% of repeat sequences classified as LTRs (Natsume et al., 2014). This analysis was based on similarity to repeats in the MIPS PlantDB (Nussbaumer et al., 2012) and MSU Plant Repeat Databases (Kohany, Gentles, Hankus, & Jurka, 2006) using RepeatMasker (Smit, Hubley, & Green, 2015). However, incompletely assembled and collapsed LTR regions obscured the true extent of repeat content in the genome. With long‐read sequencing, the challenge of assembly fragmentation can be partially overcome (Eid et al., 2009). PacBio single molecule, real‐time sequencing (SMRT) produces reads spanning an average of 10–16 kb (Ardui, Ameur, Vermeesch, & Hestand, 2018), up to nearly 100 kb (Minio, Lin, Gaut, & Cantu, 2017). In addition, algorithmic advances in long‐read assembly have given rise to phased assemblies (Minio, Massonnet, Figueroa‐Balderas, Castro, & Cantu, 2019), in which both haplotypes are assembled independently. Haplotype‐phased assemblies allow for investigation of genome structure beyond what is possible for a collapsed haploid assembly. We used long‐read sequencing to produce a high‐quality, more‐complete assembly to better resolve LTR content, regions of high heterozygosity, and gene copy number variation.

## 2 MATERIALS AND METHODS

### 2.1 Sample collection and DNA sequencing

**FIGURE 1:**
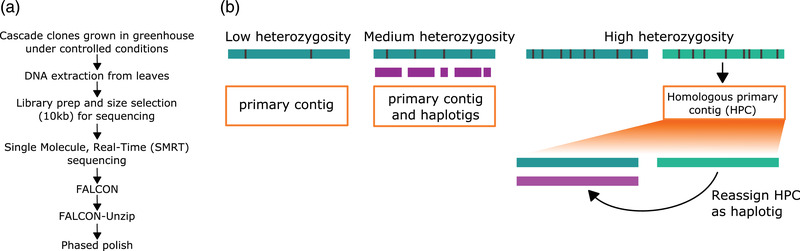
(a) Data analysis pipeline for sequencing and assembly. (b) Schematic comparing the identification of HPCs and haplotigs

To prepare for PacBio SMRT sequencing, DNA was extracted from leaf tissue (Figure 1a). Multiple samples of approximately 100 μg of young leaves from Cascade were collected and placed on ice, and DNA was extracted using a Qiagen DNeasy miniprep kit. The protocol for DNA extraction was modified to prevent shearing, including chemical precipitation and use of glass hooks instead of spin columns. Two SMRTbell libraries were constructed from DNA with a required minimum length of 10 kb. Sequencing was performed on PacBioRS II with P6‐C4 chemistry and Sequel 1.2.1‐2.0 chemistry. For the draft assembly, total raw read size was 288 Gb, 182 Gb for seed reads, and 135 Gb for the pre‐assembled reads (p‐reads), with low‐coverage regions removed during assembly. Only raw reads greater than 500 bp and p‐reads longer than 11 kb were used.

### 2.2 Genome assembly and phasing

Long‐read sequences generated by SMRT sequencing were assembled with FALCON, a diploid‐aware, de novo assembler (Chin et al., 2016). Assembly with FALCON proceeded in four stages: overlap detection and error correction of raw reads, overlap detection of error‐corrected reads, and string graph assembly. FALCON uses a hierarchical genome assembly process (HGAP) that is performed in two rounds. The first step is pre‐assembly, which involves error‐correction of raw reads. During pre‐assembly, the longest raw reads are designated as “seed reads,” which serve as the foundation for building high‐accuracy consensus sequences. Raw reads longer than 10 kb were selected as seed reads and all shorter reads were aligned to the seed reads to generate pre‐assembled reads (p‐reads), which are high‐accuracy consensus sequences. The pre‐assembled yield, defined as the total length of the p‐reads divided by the total length of the seed reads, provides a metric for assessing read quality and coverage of the p‐reads, and was 75%. During the next round of HGAP, p‐reads were aligned to each other and assembled into contigs.

Although PacBio long‐reads have a single pass error rate around 13–15% (Ardui et al., 2018), the random distribution of errors in long read sequencing permits high‐accuracy consensus sequences if sufficient coverage is available (Chin et al., 2013). Polishing of consensus sequences involves alignment of all raw reads to the draft consensus sequence contigs and further improves the quality of the final consensus sequences (Kingan et al., 2019a; Kingan et al., 2019b; Sedlazeck, Lee, Darby, & Schatz, 2018). Polishing of consensus sequences was performed with BLASR and quiver from smrtanalysis‐5.1.0. We further estimated the error rate of gene regions of our assembly (see section Calculation of Error Rates in Gene Regions).

The output assembly was phased by FALCON‐Unzip into a set of larger contigs called “primary contigs” and other, typically shorter, contigs corresponding to the haplotype called “haplotigs” (Chin et al., 2016). Phasing with FALCON‐Unzip proceeded in three stages: SNP identification and assignment of phase to reads, contig phasing, and polishing. Phasing of the haplotypes is based on the presence of structural variants (SV) and single‐nucleotide polymorphisms (SNPs) in the genome, with the level of heterozygosity determining the extent of phasing that can occur. FALCON and FALCON‐unzip were run with the November 2017 versions.

In areas of low heterozygosity, haplotypes were collapsed into a single primary contig. Areas of medium heterozygosity were used for phasing and identification of haplotigs. Highly heterozygous regions too diverged to be identified as haplotypes were assembled as independent primary contigs (Chin et al., 2016), resulting in duplicated primary contigs.

To further refine the diploid‐aware assembly, we defined the associate contigs, including both the haplotigs identified by FALCON‐Unzip and the duplicated contigs from the primary assembly that were found to correspond to the haplotype of longer primary contigs. We identified duplicated primary contigs using a variety of approaches, including purge_haplotigs (Roach, Schmidt, & Borneman, 2018) and a purely sequence alignment‐based approach. The shorter of the pair of duplicated primary contigs was assigned to the associate contigs and this subset of contigs is hereafter referred to as homologous primary contigs (HPCs) (Figure 1b).

### 2.3 Deduplication with purge_haplotigs

HPCs were identified by read coverage and sequence alignment with purge_haplotigs (Roach et al., 2018). Duplicated primary contigs were expected to have half read‐depth because reads would be split between multiple contigs. Candidate HPCs featuring half read‐depth were analyzed for homology with megablast 2.2.26 (Altschul, Gish, Miller, Myers, & Lipman, 1990), MUMmer 3.23 (Kurtz et al., 2004) and LASTZ 1.04.00 (Harris, 2007). Alignment statistics were computed to differentiate between highly repetitive alignments and homologous regions.

### 2.4 Assessment of deduplication

The deduplication strategy was assessed by Benchmarking Universal Single‐Copy Orthologs (BUSCO v2.0) (Simão, Waterhouse, Ioannidis, Kriventseva, & Zdobnov, 2015), which contains a highly‐conserved set of genes that are expected to be present in single‐copy across closely‐related organisms. BUSCO provides a measure of the extent of duplication and completeness of gene content in an assembly. The purge_haplotigs‐deduplicated assembly contained 451 duplicated BUSCO genes, suggesting that further deduplication was possible. The goal moving forward was to use BUSCO to identify which set of alignment and filtering parameters from our deduplication approach that maximized the number of single‐copy genes and minimized the number of duplicated, fragmented, and missing genes. We also ran BUSCO on the Shinshuwase and Teamaker assemblies to compare with the deduplicated assembly.

### 2.5 Sequence alignment‐based deduplication

Following deduplication with purge_haplotigs, the assembly was still larger than expected and many duplicated BUSCO genes remained. We developed a sequence alignment‐based strategy to further identify HPC pairs that resisted identification by purge_haplotigs.

The first step was to identify homologous pairs using a fast, computationally inexpensive method. Homologous regions between contigs were retrieved from megablast alignments between all primary contigs. Megablast output was sorted by score in descending order, discarding self‐hits. Top, unique hits were stored, imposing an E‐value of less than 1e‐100, while redundant, significantly overlapping hits were discarded. A position‐specific hit count along the length of the primary contig was computed, ultimately providing an average hit count for the entire length of the contig. Contigs that aligned to more than 10 contigs on average were removed from consideration as a candidate haplotype region. The hit count corresponded to a contig pair having strong homology and low occurrence of duplication (printLowDuplicationFrequencyBlastHits.py is located in the “Deduplication” directory of the CascadeHopAssembly github project). Contig pairs with a hit count above the threshold were selected for further analysis with pairwise DNA alignment tools, LASTZ and MUMmer. Visualization of MUMmer alignments with mummerplot v3.0 allowed for verification of alignment strength, as well as identification of large‐scale structural features such as tandem repeats and inversions.

To assess the strength of homology between contigs, we normalized alignment score, coverage, continuity, and percent identity for the whole alignment to get a total score or coverage relative to the length of the shorter contig. Four different density values (e.g. score density) were created using computeNonOverlappingScoreDensity.py (located in the “Deduplication” directory of the CascadeHopAssembly github project). The minimum density values for filtering were selected based on the correspondence of score or coverage density to homology between primary contigs, as validated by visual inspection with mummerplot (Supplementary Figure S1). Filtering thresholds for these density values included minimum coverage, score, continuity, and percent identity set to 20%. The maximum allowed percent overhang, defined as the maximum percentage of the shorter primary contig that remained unaligned at its beginning or end to the longer contig, was set to 40%. Identification of an HPC required an alignment between a pair of primary contigs with an overhang below this threshold.

Some contig pairs exhibited a misleadingly large score sum and score density in the presence of overlapping alignment blocks. By requiring that alignment blocks not overlap by more than 50% relative to the shorter contig, a score sum based on fewer redundant and overlapping alignment blocks was calculated.

Identification of strong homology was often confounded in the presence of short, spurious, off‐diagonal alignment blocks, resulting in inflated coverage density values. Alignment blocks from Mummer were clustered based on their proximity to remove short and distant alignment blocks. Alignment blocks were grouped into clusters if the block start positions occurred within 10 kb of each other. If the start positions were separated by more than 10 kb, a new cluster was initialized. Clusters containing five or more alignment blocks were stored to reduce the number of short, spurious, off‐diagonal hits, thereby preventing erroneous high‐coverage densities. Only clusters overlapping by less than 50% were stored. For clusters overlapping by more than 50%, the larger of the clusters was retained. Minimum cluster length was set to 20 kb.

To achieve the total coverage density of all clusters combined, the start position of the first alignment block in the cluster was subtracted from the end position of the last alignment block in the cluster, and the difference was divided by the length of the shorter contig. A cluster coverage density threshold above 25% was imposed, signifying that the total span of cluster coverage should cover at least 25% of the shorter primary contig. All values were calculated relative to the shorter contig. Once high‐confidence HPCs were identified, they were reassigned to the associate contig assembly (deduplicateContigs.py is available in the “Deduplication” directory of the CascadeHopAssembly github project). Taken together, purge_haplotigs (Roach et al., 2018) and our sequence alignment‐based deduplication strategy captured a more‐comprehensive set of HPCs (Supplementary Figure S2).

We found FALCON‐Unzip haplotigs and HPCs identified by purge_haplotigs that overlapped in position on their corresponding primary contig. The shorter of the overlapping FALCON‐Unzip haplotigs and HPCs were removed from the haplotig assembly to prevent redundancy in calculating statistics (assessHaplotigOverlap.py is located in the “HaplotigOverlapFiltering” directory of the CascadeHopAssembly github project).

### 2.6 Repeat prediction, annotation, and quantification

The primary and associate contig assemblies were masked with RepeatMasker 4.1.0 (Smit et al., 2015) using a database of eudicot repeat elements from MIPS PlantDB 9.3 (Nussbaumer et al., 2012). De novo identification of LTRs was performed with LTR_FINDER 1.0.2 (Xu & Wang, 2007), LTRharvest 1.5.9 (Ellinghaus, Kurtz, & Willhoeft, 2008), and LTR_retriever (Ou & Jiang, 2018). The resulting combined, masked assembly was used for gene prediction. Repeat content was quantified by counting the number of bases associated with each repeat type and dividing by either the size of the assembly or by the total number of bases associated with repeats in the assembly (Scripts available in “Figure3_RepeatAnalysis” directory of the CascadeHopAssembly github project).

### 2.7 SNP identification

Plant material, DNA extraction, library prep, sequencing, and identification of SNP markers for developing a linkage map were previously reported (Henning, Hill, Darby, & Hendrix, 2017b) except for using the draft haplotype‐phased assembly as reference genome. Over one million SNPs were identified across 660 unique genotypes, including both cultivars and USDA experimental varieties. Genotype‐by‐sequencing (GBS) data from 94 offspring and parents for a population study to identify alleles associated with short‐stature (dwarf mapping) was extracted from this global data set and further filtered to include markers with 2X coverage and presence in 95% of all mapping population and parents. GBS data from this data set were exported in hapmap format for linkage map development.

### 2.8 Linkage map

Estimation of a linkage map for a dwarf mapping population followed the same process as previously reported (Henning et al., 2017b). Approximately 13,000 highly‐filtered markers were identified. We developed linkage maps for both parents and then integrated both maps into a single consensus map using the same methods as previously reported. The final map contained 3,120 SNP markers across 10 linkage groups. The percent of the assembly anchored to the linkage map was calculated by summing the lengths of the primary contigs containing a marker and dividing by the total length of the assembly.

### 2.9 RNA‐seq preparation, transcriptome assembly, and differential expression analysis

RNA‐seq libraries from leaf, meristem, and stem tissues from Cascade were generated with RNeasy Plant Mini Kit (Qiagen), using glass hooks to remove RNA from tubes. Sequencing was performed on an Illumina HiSeq 3000 with 100 bp stranded, paired‐end libraries. Three TruSeq RNA (Qiagen) libraries were developed from the three tissues and each library was sequenced on a single lane. The transcriptome assembly was generated by aligning RNA‐seq to the deduplicated primary contig assembly with Hisat2 2.2.0 (Pertea, Kim, Pertea, Leek, & Salzberg, 2016), followed by assembly into transcripts with Stringtie v1.3.3b (Pertea et al., 2015). Protein‐coding sequences were predicted with TransDecoder 3.0.1 (Haas et al., 2013) and only the longest open reading frame (ORF) sequences were used for further analysis. Differentially expressed genes in gland, leaf, meristem, and stem tissues were identified with cuffdiff v2.2.1 (Trapnell et al., 2012).

### 2.10 Gene prediction

The masked, deduplicated assembly was used as a reference for gene prediction with Augustus (Stanke & Waack, 2003). Multiple sources of evidence were incorporated as hints to Augustus 3.3.2 for gene prediction (Stanke, Schöffmann, Morgenstern, & Waack, 2006) with *Arabidopsis thaliana* as the training model (Mitsui et al., 2015). Hints were derived from alignments to ESTs, Embryophyta genes from UniProt (accessed October 10, 2017), predicted protein‐coding transcripts from hop, and hop RNA‐seq. Hop ESTs from TrichOME V3 (Dai et al., 2010) and NCBI (accessed 12 Nov. 2018) were aligned to the masked, deduplicated assembly using blastn 2.10.0. Next, all ESTs with a hit to the assembly were aligned to the assembly with exonerate 2.2.0 (Slater & Birney, 2005). UniProt genes were aligned to the masked, deduplicated assembly with blastx 2.10.0, and then all UniProt genes with a hit to the assembly were aligned to the assembly with exonerate. Protein‐coding transcripts generated by TransDecoder were aligned to the masked, deduplicated assembly with exonerate.

Gene models were filtered to keep only the longest transcript per gene, requiring protein transcripts to be 150 amino acids or longer (Tiessen, Pérez‐Rodríguez, & Delaye‐Arredondo, 2012). We identified and removed transposon‐associated genes based on similarity (E‐value ≤ 10^‐5^) to a set of 91 transposon‐associated Pfam domains (Supplementary Table S2), which we collected through searching for transposon‐associated keywords in their descriptions (Pfam domains were accessed on 4 July 2018). Genes with only transposon‐associated domains, and no other domains, were removed from further analysis. Genes containing only homology to a bacteria gene from UniProt (accessed 17 Feb. 2020) were also filtered from further analysis (E‐value ≤10^‐5^; percent identity ≥50%; query coverage ≥75%). Gene models were also filtered based on the presence of external supporting evidence during gene prediction. Filtering scripts are available in the “FilterGeneModels” directory of the CascadeHopAssembly github project. Gene IDs are provided for hop ESTs from NCBI and TrichOME, Pfam, UniProt Embryophyta, and UniProt bacteria genes, as well as associated top hits to hop genes where applicable, in Supplementary Tables S3–S7.

### 2.11 Calculation of error rates in gene regions

Although PacBio long read sequencing can achieve low error rates after polishing given sufficient sequencing depth, it is important to assess the presence of errors in gene regions, which may impact gene prediction (Watson & Warr, 2019). We aimed to quantify the error rate in the predicted gene regions of our assembly with RNA‐seq alignments using the GATK4 variant calling pipeline (Van der Auwera et al., 2013). For this pipeline, we used GATK and Picard Tools programs associated with GATK version 4.0.1.1. The equation to calculate the error rate is the same as the equation to calculate the variant rate from RNA‐seq alignments. To get the error rate, we counted the total number of errors in non‐overlapping exons from full transcripts and divided by the total non‐overlapping length of all exons from full transcripts in the assembly (scripts available in the “ErrorDetection” directory of the CascadeHopAssembly github project). To prepare the set of full transcripts for error rate calculation, the transcripts were filtered to only include transcripts with protein‐coding potential, as determined by TransDecoder. This filtering was performed to restrict our analysis to higher‐confidence gene regions. Further, only transcripts with at least one non‐overlapping exon were included, resulting in 18,501 transcripts.

The first step toward identifying errors was alignment of the RNA‐seq to the reference assembly in two passes using STAR 2.7.1a (Dobin et al., 2013), requiring reads to map to one location. Both aligned and unaligned RNA‐seq reads were merged with MergeBamAlignment. Read groups were added with AddOrReplaceReadGroups, and duplicate reads were identified with MarkDuplicates. Reads were split into exon fragments with SplitNCigarReads based on the presence of Ns. Two rounds of base quality score recalibration were performed with BaseRecalibrator and ApplyBQSR. Variants were identified with HaplotypeCaller. Quality filtering was performed with VariantFiltration, requiring a minimum cluster size of three and cluster window size of 35, a minimum quality depth (QD) of 2, and a PHRED‐scaled probability of strand bias, FisherStrand (FS), set to 30 (Table 4; Supplementary Figure S6). Further detail about specific commands is included in the pipeline description in the Supplemental Materials.

### 2.12 Gene family clustering analysis

Protein sequences from 115 species covering a range of clades from the tree of life (Supplementary Table S9) were downloaded from respective sources. The sequences were provided to the gene orthology prediction workflow involving InParanoid (Sonnhammer & Östlund, 2014) as previously described (Myburg et al., 2014). Gene family clusters were queried further to find unique and overlapping genes between the five gene sets from *A. thaliana*, *V. vinifera*, *C. sativa*, as well as the primary and associate contig gene models. The unique gene clusters identified in the two hop assemblies were queried for potential GO terms (Ashburner et al., 2000), biological pathways, and InterProScan domain (Finn et al., 2016) associations (Supplementary Table S8; Supplementary Figure S7a).

### 2.13 Phylogenetic tree and co‐linearity analysis

**FIGURE 2:**
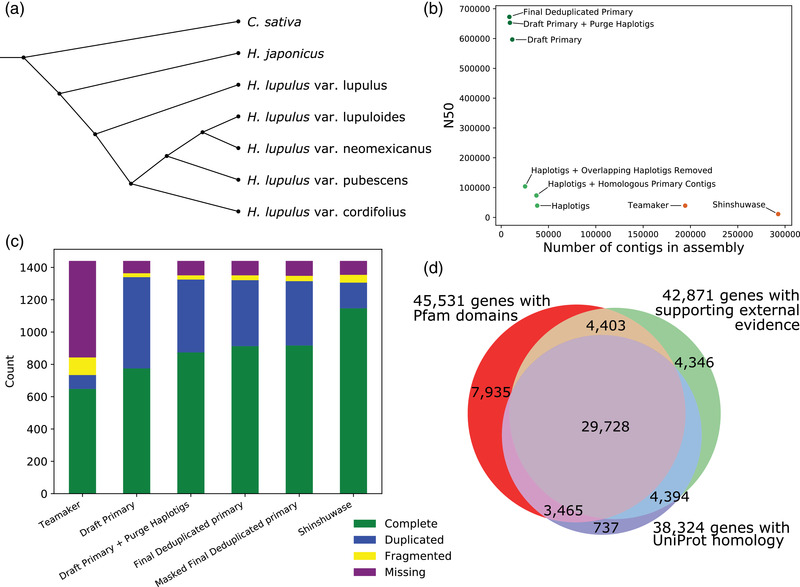
(a) Cladogram of the Cannabaceae family. (b) Scatterplot comparing N50s with number of contigs for different assemblies. (c) Stacked bar plot comparing BUSCO results for the assemblies. (d) Venn diagram comparing the number of gene models with support from external evidence during gene prediction, containing Pfam homology, and homology to UniProt genes

Figure 2a shows a cladogram of the Cannabaceae family that was derived from an analysis of North American hop lineages (Reeves & Richards, 2011) and a molecular phylogeny of wild hop samples from different continents (Murakami et al., 2006), and was constructed with FigTree v1.4.4 (Rambaut, 2012). However, the designation of the North American lineages as being species or sub‐species is debated (Tembrock, McAleer, & Gilligan, 2016).

To assess homology and evolutionary distance between hop and *C. sativa*, a phylogenetic tree was constructed (Figure 5d). First, all hop protein sequences were aligned to the set of 37,364 Embryophyta genes from UniProt with blastp 2.10.0 (E‐value ≤10^‐5^), and the same step was taken with the protein sequences from *C. sativa* (Grassa, 2018). Significant hits to cannabidiolic acid synthase (CBDAS and CBDAS‐like) genes, berberine bridge enzyme‐like genes (BBE‐like), and tetrahydrocannabinolic acid synthase (THCAS and THCAS‐like) genes were extracted for both hop and *C. sativa*, and these hits were aligned to each other. A multiple sequence alignment (MSA) was performed on this set of genes with clustalw2 2.1 (Thompson, Higgins, & Gibson, 1994). Gapped regions of the MSA were removed with trimAL v1.4.rev22 (Capella‐Gutiérrez, Silla‐Martínez, & Gabaldón, 2009). A maximum likelihood phylogenetic tree was constructed from the top hits between hop and *C. sativa* genes using the BLOSUM62 substitution matrix with PhyML 20120412 (Guindon et al., 2010). The tree was visualized using the python module ETE3 (Huerta‐Cepas, Serra, & Bork, 2016). Scripts are available in the “Figure5_Colinearity” directory of the CascadeHopAssembly github project.

Mutual best hits (MBHs) were identified from the set of hop and *C. sativa* protein sequences with a significant hit to CBDAS, CBDAS‐like, THCA, THCA‐like, and BBE‐like genes. Several co‐linear regions featuring genes from chromosome nine of the high‐CBDA cultivar were investigated. Co‐linearity was visualized with Integrative Genomics Viewer (IGV) 2.4.3 (Thorvaldsdóttir, Robinson, & Mesirov, 2013). Scripts are available in the “CannabisComparison” directory of the CascadeHopAssembly github project.

The extent of gene duplication among hop and *C. sativa* genes was assessed by counting the number of times a UniProt gene featured significant homology to a hop or *C. sativa* gene. An enrichment value was computed by subtracting the number of *C. sativa* hits from the number of hop hits, and then dividing by the number of *C. sativa* hits plus one. The enrichment value provided a measure of the relative abundance of gene copy number variation between hop and *C. sativa* (Supplementary Figure S8).

### 2.14 Analysis of coding sequences

To assess divergence between the genes of primary and associate contigs, we developed an approach for extracting gene sequences from the alignment blocks of primary and associate assemblies. We first aligned the primary and associate contigs with LASTZ, and then extracted and concatenated exon sequences, including gaps, from both primary and associate contig alignment blocks based on the coordinates of the gene model in the primary contig. Gene model coordinates in the primary contigs were projected to the corresponding positions in the associate contig alignment blocks (buildMAFIndex.py, getAlignmentSequenceFromMAF.py, and splitFasta.py are available in the “ExtractCDS” directory of the CascadeHopAssembly github project). CDS alignments were processed with MACSE v2.03 (Ranwez, Harispe, Delsuc, & Douzery, 2011) using the exportAlignment option. Internal stop codons were denoted as ‘NNN’ and codons containing frameshifts were denoted as ‘—’. Further processing was performed to remove frameshift‐containing and stop codons (processExportAlignmentOutFile.py is in the “ExtractCDS” directory of the CascadeHopAssembly github project).

### 2.15 Large‐scale structural variation between haplotypes

Contig‐scale variation between primary and associate contigs was visualized with mummerplot. Gap lengths, Kimura 80 (K80) distances (Kimura, 1980), and conditional probabilities were calculated from the alignment blocks of primary and associate contigs generated by LASTZ. Additionally, K80 distance was calculated for other primary pairs that featured weak homology, but did not meet the threshold for being classified as HPCs. Scripts are available in the “Figure6_StructuralAnalysis” directory of the CascadeHopAssembly github project.

### 2.16 Gene‐scale variation between haplotypes: Kimura 80 distance and assessment of GO term enrichment

We assessed and quantified variation between haplotypes at the gene level by calculating the K80 distances between primary and associate gene pairs, which were extracted using the method described above in “Analysis of Coding Sequences.” Predicted gene function was assigned by aligning the primary assembly genes to Embryophyta genes from UniProt. GO terms were assigned based on similarity to associated UniProt genes.

We then assessed the functional enrichment of highly diverged genes in HPCs. To investigate functional enrichment of highly diverged HPC genes, we first identified haplotig genes with the top 5% most diverged K80 distances. The smallest distance in the top 5% of K80 distances was used to set the K80 distance threshold for HPC genes. We performed a binomial test to assess whether GO term enrichment of genes in HPCs with a K80 distance above the distance threshold was significantly greater than the expected rate of GO term enrichment among haplotig genes with the top 5% of K80 distances. Probability *p* was calculated from the full set of primary genes with a GO term, while *k* was the observed occurrence of a given GO term in the HPCs, and *n* was the total number of genes with an associated GO term above the K80 distance threshold (Supplementary Figure S12). The expected probability for HPC GO term association was calculated by multiplying *n* and *p*, and the binomial test was performed only on GO terms with an observed *k* above the expected probability. We also performed a beta‐binomial test to account for overdispersion of the probability. To estimate the parameters for the beta‐binomial, we first generated 10,000 random gene lists the same size of the HPC gene set from the full set of primary assembly genes and calculated the mean and variance for each GO term probability. Alpha and beta for each GO term were calculated from the average mean and variance. Scripts are available in the “GeneAnalysis” directory of the CascadeHopAssembly github project.

## 3 RESULTS

The goal of a phased diploid‐aware assembly is to create two assemblies corresponding to the two haplotypes: a primary and an associate assembly. We were motivated to create a draft, phased, long‐read assembly to assess and quantify genomic variation between haplotypes, while also assembling larger contigs to enable the comparison of gene content to other members of the Cannabaceae family, including *Cannabis sativa* and *Humulus japonicus* (Figure 2a). Our long‐read PacBio assembly represents an intermediate product in the progression towards a chromosome‐level assembly, and here we report gene and repeat content for this improved draft assembly.

The initial primary assembly was 4.24 Gb, containing 11,705 contigs, and the initial associate assembly was 1.35 Gb, containing 38,060 haplotigs (Table 1). Following deduplication with purge_haplotigs, 2,491 of the 11,705 primary contigs were identified as HPCs. However, the 3.82 Gb primary assembly was still far larger than the true estimated genome size (2.8 Gb), which was not unexpected given its high heterozygosity (McCartney et al., 2019).

**TABLE 1:** Assembly statistics

Metric	Draft primary	purge_haplotigs	Deduplicated primary	Draft associate	Associate (no overlap filtering)	Associate (overlaps filtered)	Shinshuwase	Teamaker
Assembly length	4.24 Gb	3.82 Gb	3.71 Gb	1.35 Gb	1.78 Gb	1.49 Gb	1.81 Gb	2.77
Number of contigs	11705	9214	8661	38060	37223	25239	292698	194438
N50	596.74 kb	652.97 kb	672.60 kb	39.43 kb	73.50 kb	103.97 kb	11.13 kb	39.33 kb
Largest contig	8.25 Mb	8.25 Mb	8.25 b	1.77 Mb	1.77 Mb	1.77 Mb	129.33 kb	367.72 kb

We developed a pairwise, sequence alignment‐based approach to further identify and reassign HPCs. This involved collecting pairs of primary contigs with strong similarity and then reassigning the shorter primary contig as an HPC. A variety of alignment filtering parameters, such as score density, were used to identify HPCs (Supplementary Figure S2). We identified an additional 568 HPCs, reducing the primary assembly to 3.71 Gb (8,861 contigs).

Following reassignment of HPCs, the size of the associate assembly increased to 1.78 Gb (37,223 contigs). The N50 of the draft primary assembly was 596.74 kb, and the N50 of the primary assembly following deduplication with purge_haplotigs was 652.97 kb. Ultimately, the N50 of the final, deduplicated primary assembly was 672.6 kb. The N50 of the draft haplotig assembly was 39.43 kb, while the N50 of the haplotig assembly following reassignment of HPCs was 73.5 kb (Figure 2b; Table 1).

Many haplotigs and HPCs identified by purge_haplotigs overlapped in position on their corresponding primary contig. The shorter of the overlapping haplotigs and HPCs were removed from the associate assembly to prevent redundancy in downstream analyses. Following overlap removal, the N50 of the haplotig assembly increased to 103.97 kb. Haplotigs comprise 65.88% of the associate assembly and cover 26.5% of the primary assembly, while HPCs comprise 34.12% of the associate assembly and cover 13.73% of the primary assembly (Table 2).

**TABLE 2:** Associate assembly percentage of coverage

Percentage assembly coverage	Deduplicated primary	Associate
Haplotig (overlap unfiltered)	33.7	70.3
HPC (overlap unfiltered)	14.24	29.7
Haplotig (overlap filtered)	26.5	65.88
HPC (overlap filtered)	13.73	34.12

The final, deduplicated assembly was chosen based on reduction of assembly size and most improved BUSCO results (Figure 2c; Supplementary Table S1). For BUSCO assessment, our goal was to maximize the number of single‐copy complete genes, while minimizing the number of duplicated and missing BUSCO genes. The final, deduplicated assembly recovered 1,321 out of 1,440 BUSCO genes (91.7%). Out of 1,321 complete BUSCO genes, 913 are single‐copy (63.4%) and 408 are duplicated (28.3%). In contrast, the purge_haplotigs‐deduplicated primary assembly contained 874 single‐copy and 451 duplicated BUSCO genes.

We also compared the BUSCO results for our assembly with the Shinshuwase and Teamaker assemblies (Figure 2c). Although the Shinshuwase assembly contains more single‐copy, complete BUSCO genes (1,147) and fewer duplicated BUSCO genes (159), the assembly is highly fragmented, given the large number of scaffolds in the assembly (292,698), low N50 (11.13 kb) (Figure 2b; Table 1), and short scaffold length (Supplementary Figure S3). The relatively low repeat content of the Shinshuwase assembly suggests that scaffolds were broken in repeats and that repeats are collapsed in the assembled contigs. The fragmented composition of the short‐read assemblies, as demonstrated by low N50 and large number of short scaffolds, suggests they are composed of gene islands and do not comprehensively capture intergenic and transposon regions. The Shinsuwase assembly contains 22,201 annotated protein‐coding genes (Natsume et al., 2014) and the Teamaker assembly contains 16,161 annotated protein‐coding genes (Hill et al., 2017) after removing repeat‐associated protein‐coding genes.

Gene prediction with Augustus (Stanke & Waack, 2003) generated 153,703 gene models in the primary assembly and 65,274 gene models in the associate contig assembly. Requiring a length of at least 150 amino acids resulted in 91,258 gene models in the primary assembly. Of the 91,258 genes in the primary assembly, 486 genes feature homology only to a bacterial gene from UniProt (Table 3). There were 9,168 genes featuring homology only to a repeat‐associated domain and 5,843 genes featuring homology to both repeat‐ and non‐repeat‐associated domains in the primary assembly (Table 3). Genes with homology to only a repeat‐associated Pfam domain were removed from further analyses. After filtering gene models based on homology to repeat‐associated Pfam domains and bacterial genes, 81,604 gene models remained in the primary assembly and 31,120 gene models remained in the associate contig assembly (Table 3). In the primary assembly, 42,871 genes had supporting external evidence during gene prediction from RNA‐seq transcripts, ESTs, or UniProt Embryophyta genes.

**TABLE 3:** Gene prediction and annotation

Number of genes	Deduplicated primary	Associate (haplotigs + HPCs)
Total unfiltered	153,703	65,274
Genes encoding proteins with length above 150aa	91,258	34,625
Genes with significant homology to UniProt bacteria gene	486	184
Gene predictions supported by evidence	42,871	5,302
Genes with Pfam domain	45,531	18,296
Genes with a repeat‐associated Pfam domain	9,168	3,321
Genes with repeat and non‐repeat‐associated domains (not removed from assembly)	5,843	2,344
After removing repeat‐ and bacteria‐associated genes	81,604	31,120
Significant hit to UniProt (no repeat filtering applied)	41,092	12,484
Significant hit to UniProt (repeat filtering applied)	38,324	11,868
Non‐redundant hit to UniProt (no repeat filtering applied)	10,338	5,712
Non‐redundant hit to UniProt (repeat filtering applied)	10,321	5,697

There are 38,324 gene models in the primary assembly featuring significant similarity to a UniProt gene and 10,321 of those hits to UniProt are unique, suggesting widespread occurrence of paralogs (Table 3). Overall, 29,728 gene models are supported by external evidence and feature homology to both Pfam domains and UniProt Embryophyta genes (Figure 2d). Over 12,000 gene models unsupported by external evidence during gene prediction contain homology to a Pfam domain or a UniProt gene. We used the full set of genes, including models unsupported by external evidence, while excluding repeat‐associated genes and bacteria‐associated genes, for homology analyses. Genes without external evidence were excluded from the K80 distance analyses to limit our comparison to higher‐confidence gene models.

We assessed the error rate in gene regions by aligning RNA‐seq to the assembly and identifying substitutions and indels with the GATK4 variant calling pipeline (Van der Auwera et al., 2013). The average substitution rate for all tissues ranges from 0.19–0.22. The average indel rate for all tissues ranges from 0.07–0.08% (Table 4; Supplementary Figure S6).

**TABLE 4:** Percentage of error rate in gene sequences

Tissue and replicate	Average substitution error rate	Average indel error rate
	%	
Leaf replicate 1	0.1891	0.068
Leaf replicate 2	0.1888	0.068
Meristem replicate 1	0.2224	0.0824
Meristem replicate 2	0.2222	0.0822
Stem replicate 1	0.2084	0.0763
Stem replicate 2	0.2102	0.07772

**FIGURE 3:**
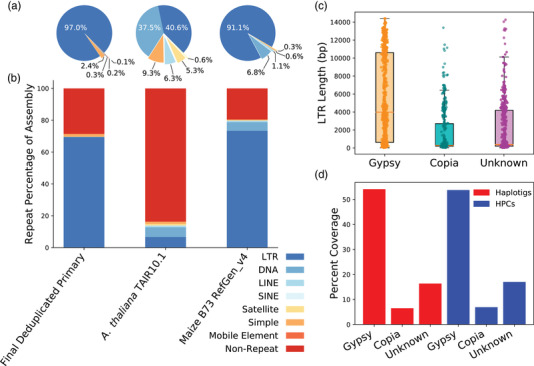
Repeats were identified with a de novo library of LTR sequences and a database of eudicot non‐LTR repeat elements from MIPS PlantDB. (a) Pie chart comparing repeat percentages relative to total repeat content in our deduplicated primary assembly with *A. thaliana* and maize. The percentage for each repeat type was quantified by counting the total number of repeat‐associated bases for that type in the assembly, divided by the total number of repeat‐associated bases. (b) Stacked bar chart depicting repeat percentages relative to assembly length in our deduplicated primary assembly, *A. thaliana*, and maize. (c) Length distribution of LTR sequences. (d) Bar chart showing percent coverage of the three LTR sub‐types in haplotigs and HPCs. The percentage for each repeat type was quantified by counting the total number of repeat‐associated bases for that type in the assembly, divided by the size of the assembly

We identified 1,679 non‐redundant Gypsy, Copia, and unknown‐type retrotransposons with a de novo approach, using LTR_FINDER (Xu & Wang, 2007), LTRharvest (Ellinghaus et al., 2008), and LTR_retriever (Ou & Jiang, 2018). We then combined the set of de novo LTR sequences with the repeat database from MIPS PlantDB (Nussbaumer et al., 2012) and annotated the assembly with RepeatMasker (Smit et al., 2015). We then quantified the repeat content of the deduplicated genome (Figure 3). We found that LTRs comprise 97.04% of total repeat content by length, comparable to maize (91.14%) and over twice as much as *Arabidopsis* (40.57%) (Figure 3a). When calculated as a proportion of the total genome length, the deduplicated assembly is 71.46% repetitive (Figure 3a,b; Table 5). Gypsy‐type elements are the longest type of LTR in hop, with an average length of 4 kb, and are also the most abundant (Figure 3c). Given the high repeat content of the hop genome, we investigated whether repeat content was responsible for the greater heterozygosity in HPCs compared to haplotigs. We found that both types of associate contig contain a similar percentage of repeat content (Figure 3d).

**TABLE 5:** Percentage of assembly covered by repeats

Category	Deduplicated primary	Associate (overlaps filtered)
Total	71.46	78.50
LTR	69.35	76.53
DNA	0.21	0.21
LINE	0.04	0.04
SINE	0.00	0.00
Simple	1.70	1.57
Mobile element	0.15	0.13
rRNA	0.01	0.01
Other	0.02	0.02
Non‐repeat	28.54	21.50

**FIGURE 4:**
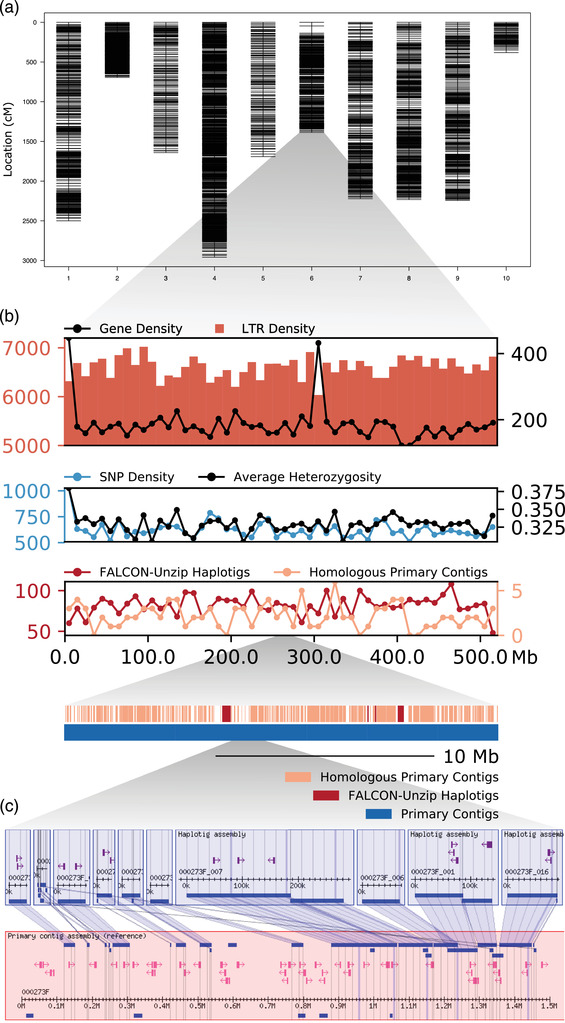
(a) Linkage groups built from the deduplicated primary assembly and a dwarf mapping population containing 3,120 SNPs. (b) Average heterozygosity, SNP density, gene density, and LTR density in linkage group 6. (c) Visualization of haplotype assembly alignment blocks from primary contig 000273F and its associate contigs with Gbrowse_syn

We estimated a linkage map using a dwarf mapping population (Henning et al., 2017b) containing 3,120 SNP markers in 10 linkage groups (Figure 4a). The linkage map is anchored by 35.6% of the primary assembly (1.32 Gb). Using the linkage groups, we mapped associate contigs, heterozygosity, SNP, gene, and LTR density to map‐ordered primary contigs to visualize large‐scale structural patterns and densities (Figure 4b). All of linkage group 6 is presented in Figure 4, depicting a region of over 500 Mb. At the scale of 10 Mb, we observed two regions in linkage group 6 where spikes in gene density corresponded to depleted LTR density. We calculated the genome‐wide correlation between genes and LTRs to assess if this region of inverse abundance was indicative of a genome‐wide pattern. However, on a genome‐wide scale, gene and LTR density are correlated across 10 Mb windows (Pearson correlation 0.757, p‐value 0) (Supplementary Figure S4). To visualize structural variation between primary and associate contigs, we used Gbrowse_syn (Figure 4c). We also visualized the association between genetic and physical positions (Supplementary Figure S5).

We investigated the association of protein‐coding genes in the primary and associate contig assemblies with protein‐coding genes from 115 species representing a range of clades from the tree of life (Supplementary Figure S7a; Supplementary Tables S8 and S9) clustered in 117,088 gene families. A comparison of 21,014 gene family clusters containing at least one of the genes from *A. thaliana*, *V. vinifera*, *C. sativa*, as well as the primary and associate contig assemblies, revealed that ∼90% of associate contig genes and 82% of genes from the primary assembly have gene family assignments, as compared to 93% of *A. thaliana* and ∼84% of genes from *C. sativa* and *V. vinifera*. Between the five genome assemblies, there were 3,462 common gene families. We also found that 1,054 and 2,154 genes were unique to the associate and primary contig assemblies, present in 577 and 1,201 gene families, respectively (Supplementary Figure S7a). More than half of these gene families contain more than one hop gene family member, while the rest are shared with genes from the remaining species.

Of the 18,835 repeat‐filtered primary genes that overlap haplotigs, 8,071 feature a significant hit to a UniProt gene, but only 4,121 of those significant hits are unique. There are 7,604 genes overlapping HPC regions, and while 3,278 of those genes have a significant hit to UniProt, only 2,038 of the UniProt genes are unique, suggesting widespread duplication of gene function in both cases (Supplementary Table S10). Primary genes overlapping HPCs regions are enriched for single‐copy hits to UniProt (Supplementary Figure S7b).

**FIGURE 5:**
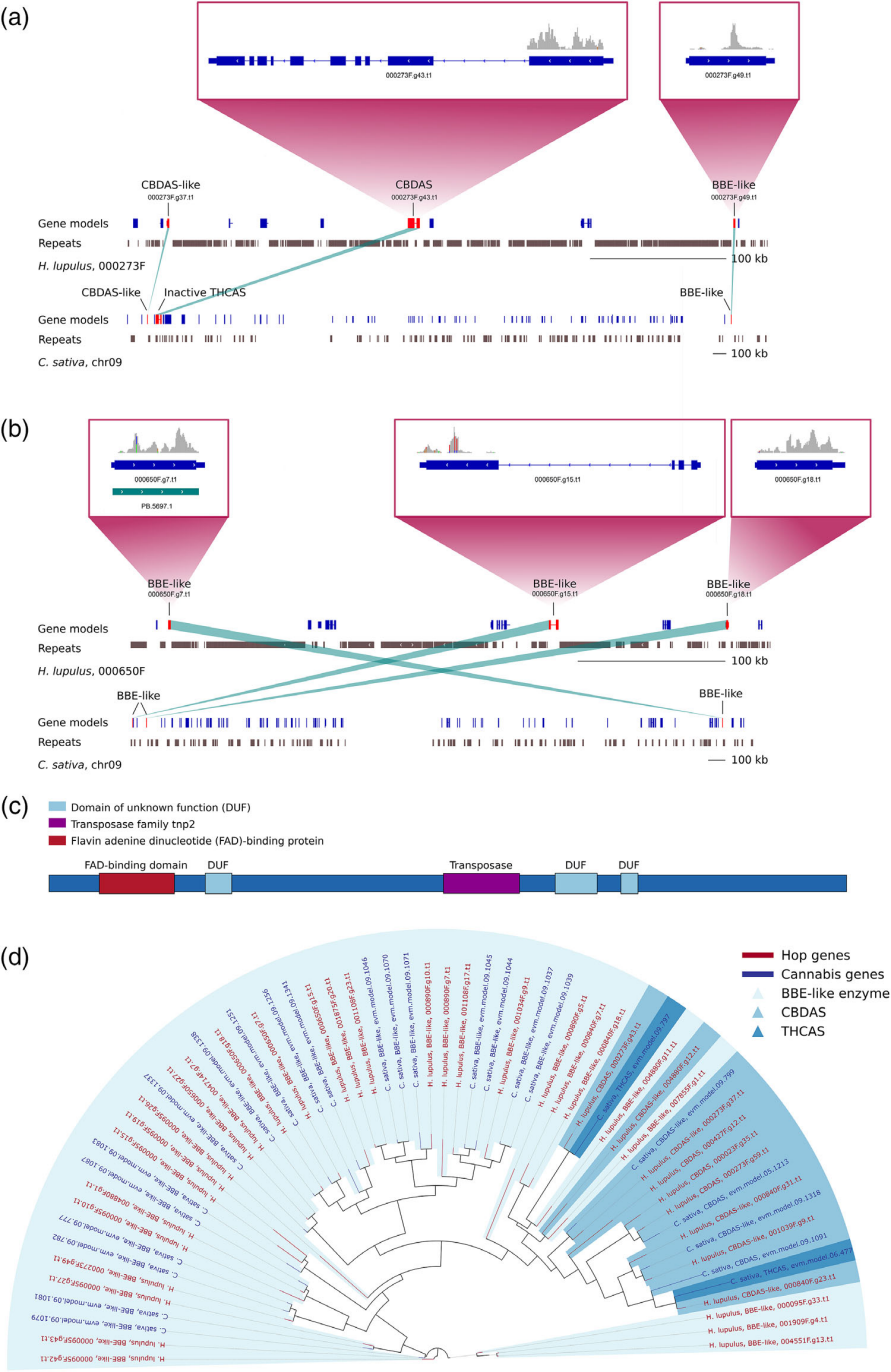
(a) Co‐linearity plot comparing genomic regions containing CBDAS homologs in hop and *C. sativa*. (b) Genomic region depicting colinear arrangement of BBE‐like enzyme homologs in hop and *C. sativa*. (c) Protein domain structure of putative CBDAS hop gene. (d) Phylogenetic tree comparing all CBDAS, THCAS, and BBE‐like enzyme homologs in hop and *C. sativa*

One of the genes containing both repeat‐ and non‐repeat‐associated domains is a putative cannabidiolic acid synthase (CBDAS) gene. The putative CBDAS gene contains three domains of unknown function (DUF), a transposase domain, and an FAD‐binding domain (Figure 5c). Only the region of the gene overlapping the FAD‐binding domain, bearing homology to CBDAS, shows evidence of expression (Figure 5a). Upstream of the expressed region of the gene, an intron contains segments of Gypsy‐type LTRs. Further upstream where the transposon domain occurs, however, no repeats are annotated.

Hop and *C. sativa* share localized regions of conserved co‐linearity of genes involved in cannabinoid synthesis (Figure 5a). Multiple putative BBE‐like, CBDAS, and CBDAS‐like hop genes share conserved co‐linearity with genes on chromosome nine of *C. sativa* (Figure 5a,b), which contains tandemly repeated CBDAS genes nested within LTRs (Grassa et al., 2018). We also identified a region of co‐localized genes featuring BBE‐like genes (Figure 5b). In total, we identified co‐localized regions of genes within two linkage groups in hop and three chromosomes of *C. sativa* (Grassa et al., 2018).

Co‐linearity was assessed by performing a MBH analysis to identify genes in hop and *C. sativa* with highest shared homology to CBDAS, CBDAS‐like, THCAS, THCAS‐like, and BBE‐like genes. Additionally, to investigate the extent of gene duplication in hop and *C. sativa*, we computed the number of occurrences of significant homology to Uniprot Embryophyta genes. We found that while *C. sativa* is enriched for single‐copy genes, hop shows enrichment relative to *C. sativa* for two and four gene copies (Supplementary Figure S8), providing preliminary supporting evidence for WGD in hop.

**FIGURE 6:**
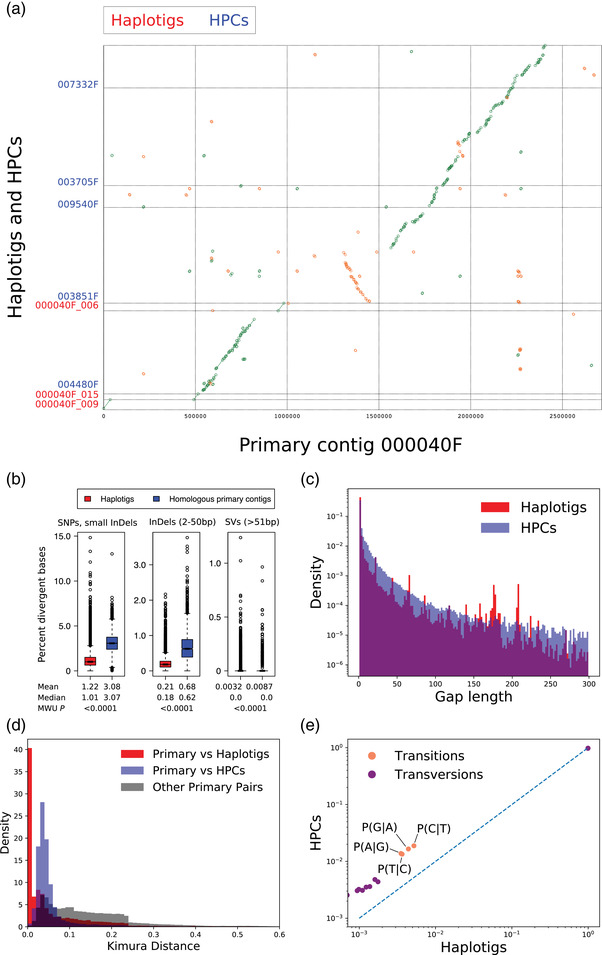
(a) Mummerplot comparing primary contig 000040F and its haplotigs and HPCs. (b) Boxplots comparing sequence divergence of haplotigs and HPCs. (c) Histogram comparing gap length distributions for haplotigs and HPCs. (d) Histogram comparing K80 distance for alignment blocks in haplotigs and HPCs, as well as other primary pairs that contained homology but did not meet the threshold to be reassigned as an HPC. (e) Scatterplot comparing the conditional probabilities of nucleotides in haplotigs and HPCs

Large‐scale structural variation between primary contigs and their associate contigs was visualized with mummerplot (Figure 6a; Supplementary Figure S9a). Three out of eight of the associate contigs corresponding to primary contig 000040F are HPCs (Figure 6a) containing structural variation, including inversions (HPCs 003851F and 003705F). Primary contig 000145F has 14 associate contigs, and all but one are haplotigs. The single HPC (004045F) covers the middle of the primary contig, and features more variation and shorter alignment blocks than the haplotigs (Supplementary Figure S9a). We observe that HPCs have greater structural variation than haplotigs, which are generally shorter than HPCs and are strongly homologous to their primary contig. Overall, alignment block coverage is larger in haplotigs than in HPCs (Supplementary Figure S9b).

SNPs, insertions, deletions, and structural variation are more abundant in HPCs than haplotigs (Figure 6b), and gap lengths are longer in primary contig and HPC alignment blocks (Figure 6c). We assessed sequence divergence between haplotypes using K80 distance (Kimura, 1980), an evolutionary distance that is a function of the rate of transitions and transversions. We did not use the K80 distance to identify HPCs. Instead, the K80 distance was used to validate the hypothesis that HPCs have greater sequence divergence than haplotigs. The K80 distance is larger between primary contigs and HPCs than primary contigs and haplotigs.

We also calculated the K80 distance for other primary pairs that did not meet the threshold for being classified as HPCs, and found overall that these pairs are more diverged than primary contigs and HPCs. This finding demonstrates that the set of HPCs we identified had greater divergence than haplotigs and lower divergence than remaining primary contigs (Figure 6d). Although HPCs are more diverged than haplotigs (Figure 6d) overall, gene regions do not adhere to this trend, with haplotigs containing more highly diverged gene pairs. The conditional probability associated with transitions is higher in the alignment blocks of primary contigs and HPCs than primary contigs and haplotigs (Figure 6e). We also found a weak correlation between the heterozygosity evaluated from 660 individuals and K80 distance between haplotypes (Supplementary Figure S10).

Based on the lack of discernible difference in the LTR content of HPCs and haplotigs, we investigated divergence due to gene content. Our goal was to assess divergence between the genes of the haplotype assemblies to establish if gene regions are responsible for the greater divergence in HPCs. We also aimed to identify biological significance related to the greater divergence observed in HPCs. To this end, we looked at the functional enrichment of the most highly diverged genes.

We compared sequence divergence of genes in the primary and associate contigs by calculating the CDS length differences and K80 distances between pairs extracted from the alignment blocks. We calculated the length difference between CDS of primary and associate contigs, which revealed a set of ∼5,000 genes without gaps. We also observe a slight periodicity of three base pairs in the length difference (Supplementary Figure S11).

We then investigated functional enrichment of genes overlapping HPCs that have the greatest K80 distance, by restricting the analysis to genes with sequence divergence greater or equal to the top 5% of distances among haplotig genes. Our results from evaluating GO terms using binomial and beta‐binomial tests show that 180 genes in the HPCs are statistically enriched for biological functions, including 30 genes associated with defense response (GO:0006952) (Supplementary Table S11). Multiple clathrin‐related GO terms are represented in the set of cellular components GO terms (GO:0030130, GO:0030132, GO:0071439). Clathrin is a protein involved in cellular signaling and uptake of nutrients (Royle, 2006). Enriched molecular function GO terms include 26 genes associated with ADP binding (GO:0043531) and 66 genes associated with ATP binding (GO:0005524). Most of the genes associated with ADP and ATP binding have high sequence similarity to disease resistance genes from UniProt.

## 4 DISCUSSION

The large size and heterozygosity of the hop genome challenged previous efforts at assembly. As a result, many questions about the hop genome have remained unanswered. In this study, we describe how we developed a draft haplotype‐phased assembly to overcome these challenges by quantifying repeat and gene content, gene duplication, and structural variation between haplotypes. We have presented a deduplication strategy to further identify and reassign HPCs beyond what was possible with limited available tools. The result is a partially‐phased, diploid‐aware assembly containing a comprehensive repertoire of genes and repeat sequences. The draft haplotype‐phased assembly offers a unique opportunity to study structural variation between the haplotype assemblies and provides insight into the evolution of hop.

Although the two types of associate contig have differing levels of sequence divergence in the form of insertion‐deletions and larger regions of structural variation, we found that the divergence cannot clearly be attributed to either gene or repeat content. Predicted genes in both haplotigs and HPCs are enriched for functions associated with growth, flowering, and stress response, but we did not observe patterns of functional enrichment that distinguish them. Therefore, despite HPCs having greater overall structural variation than haplotigs, HPCs and haplotigs are comparable in terms of their repeat content and gene function.

Hop and *C. sativa* diverged ∼27 million years ago (Divashuk, Alexandrov, Razumova, Kirov, & Karlov, 2014; Murakami et al., 2006). The genome of hop is three times larger than *C. sativa* (Natsume et al., 2014; Pisupati et al., 2018), and the significant difference in genome size is hypothesized to be a result of WGD (Matthews, Coles, & Pitra, 2013). The genome of hop is twice as large as *Humulus japonicus*. Our finding suggests that hop and *C. sativa* contain a similar percentage of repeat content, as *C. sativa* is ∼73% repetitive (Laverty et al., 2019). The similar repeat percentage between hop and *C. sativa* supports the hypothesis that hop genome expansion is primarily driven by WGD followed by a return to a diploid state through rearrangement and gene loss (Dodsworth, Chase, & Leitch, 2015; Matthews et al., 2013), rather than LTR‐driven expansion observed in other plants (Kim et al., 2014). Hop is enriched for two and four gene copies relative to *C. sativa* (Supplementary Figure S8), supporting the WGD hypothesis. However, a full understanding of the mechanism underlying the potential occurrence of WGD remains to be uncovered.

Hop and *C. sativa* share common enzymes, including polyketide synthases and prenyltransferases, that participate in the synthesis of key compounds (Marks et al., 2009). One of the hop genes containing both repeat‐ and non‐repeat‐associated domains has significant homology to CBDAS (Figure 5a). The putative CBDAS gene in hop has greater sequence similarity to CBDAS in *C. sativa*, than to BBE‐like genes in hop, supporting the idea that the putative CBDAS gene in hop is not a BBE‐like gene. This predicted gene contains external evidence for only one of the 10 exons. As shown in Figure 5a, only the first exon, which has strong homology to CBDAS is expressed, suggesting the fusion could be an artifact of gene prediction. It is expressed in lupulin gland, leaf, and meristem tissues, with highest expression occurring in leaf.

Although gene prediction was performed on the masked assembly, repetitive regions may have been incompletely masked, which might explain why prediction extended through a repeat sequence. The predicted fusion of a transposon domain and putative CBDAS gene in hop is a telling consequence of the highly repetitive composition of the genome, and is consistent with a previous finding that CBDAS genes are located within LTR regions in *C. sativa* (Grassa et al., 2018; Laverty et al., 2019).

CBDAS is the hypothesized common ancestor of CBDAS and THCAS (Onofri, de Meijer, & Mandolino, 2015) and both genes share high sequence similarity (84%) (Taura et al., 2007). The presence of CBDAS in hop further supports the hypothesis that CBDAS is the ancestral gene of CBDAS and THCAS because CBDAS would be present in the common ancestor. The emergence of THCAS is thought to be a result of CBDAS duplication and diversification (Onofri et al., 2015), possibly copied and dispersed by LTRs (Grassa et al., 2018). Both synthases also share high sequence similarity with BBE‐like enzymes, which catalyze oxidation reactions and are highly abundant in plants (Sirikantaramas et al., 2004). To our knowledge, we are the first to report the presence of CDBAS in hop. However, genes with strong homology to CBDAS and THCAS have been predicted in plant species besides *C. sativa*, as indicated by sequences available on NCBI. Further work will be necessary to refine the gene models containing both repeat and non‐repeat‐associated domains, to assess the legitimacy of gene models containing partial external evidence, and to establish the role of the CBDAS homolog in hop.

After removing repeat‐ and bacteria‐associated genes, there were 81,604 genes in our primary assembly, with 42,871 supported by external evidence during gene prediction. Recent assemblies of barley (∼5.3 Gb), *Aegilops tauschii* (4.3 Gb), and *Nicotiana tabacum* (4.5 Gb) each contained >80,000 gene models prior to filtering for high‐confidence genes (Luo et al., 2017; Mascher et al., 2017; Sierro et al., 2014). Similarly, filtering our gene set for sequence homology and protein domains results in a core set of nearly 30 thousand genes (Figure 2d). Future work will involve integrating gene models across assemblies to produce a refined collection of hop genes, and providing a mapping of genes between the different assemblies.

HPCs correspond to genomic regions with overall greater sequence variation compared to haplotigs, and we observe a greater sequence divergence at the gene level. Genes with greater sequence divergence in the HPCs tend towards flowering, growth, and response to abiotic and biotic stresses. This suggests some gene regions within the HPCs are impacted by shifting abiotic and biotic stress conditions, a possible reaction to the unique pressures of cultivation that separate the English‐Russian maternal pedigree from the USDA paternal pedigree of Cascade.

Our deduplicated assembly also lays a foundation for chromosome‐level scaffolding with a high‐throughput chromatin conformation capture (Hi‐C) library, which will further refine the assembly by providing long‐range DNA contact information. In the primary assembly, 64.4% (2.39 Gb) was not anchored to the linkage map, indicating that further scaffolding is necessary to map the order and orientation of contigs. Readers should take into consideration that the long‐range structural integrity of this draft assembly, including the presence of chimeric contigs, has not been evaluated. Going forward, this draft assembly will provide the basis for a chromosome‐level assembly to further investigate the occurrence of WGD in hop (Pisupati et al., 2018), and understand how duplication, repeat‐expansion, and conserved synteny have driven biosynthesis and disease resistance in hop.

We have provided our approach to deduplicate the draft phased primary assembly, annotate genes and repeats, and analyze variation between the haplotype assemblies of the hop genome. Our analysis includes an updated estimation of repeat content and a more‐comprehensive set of draft gene models, as well as an investigation of functional enrichment of the most diverged genes in the HPCs and the extent of gene duplication. We expect that our draft haplotype‐phased assembly will provide a foundational resource for the Cannabaceae genomics community and for future efforts in hop research.

## CONFLICT OF INTEREST STATEMENT

SK, PP, GC, and DR are full‐time employees at Pacific Biosciences, a company developing single‐molecule sequencing technologies.

## FUNDING

Funding for sequencing was provided by PacBio and Sierra Nevada Brewing Company. This work was supported by a grant from USDA (USDA‐ARS CRIS Project 2072 21000 051 00D). LKPC is supported by an AFRI Predoctoral Fellowship (grant no. 2020‐67034‐31722) from the USDA National Institute of Food and Agriculture. JE and PJ are partially supported for their gene family work by NSF USA award #IOS:1340112.

## Supporting information



Supplementary Material

Supplementary Material

Supplementary Material

Supplementary Material

Supplementary Material

Supplementary Material

Supplementary Material

Supplementary Material

Supplementary Material

Supplementary Material

Supplementary Material

Supplementary Material

## Data Availability

The datasets generated and analyzed in this study are available at http://hopbase.org and NCBI under the BioProject ID PRJNA562558. Scripts are available at: https://github.com/padgittl/CascadeHopAssembly/.
