# Supplementary material for: A draft phased assembly of the diploid Cascade hop (Humulus lupulus) genome
Source: Plant Genome. 2021 Feb 18;14(1):e20072. doi: 10.1002/tpg2.20072 (PMC12807344; doi:10.1002/tpg2.20072)
Supplement: Supplementary file 1 [file TPG2-14-e20072-s002.docx]

Supplementary Materials for A Draft Phased Assembly of the Diploid Cascade Hop (*Humulus lupulus*) Genome

Lillian K. Padgitt-Cobb^1^, Sarah B. Kingan^2^, Jackson Wells^3^, Justin Elser^6^, Brent Kronmiller^3^, Daniel Moore^4^, Gregory Concepcion^2^, Paul Peluso^2^, David Rank^2^, Pankaj Jaiswal^6^, John Henning^4^*, David A. Hendrix^1,5^*

1 Department of Biochemistry and Biophysics, Oregon State University

2 Pacific Biosciences of California,

3 CGRB, Oregon State University

4 USDA ARS

5 School of Electrical Engineering and Computer Science, Oregon State University

6 Department of Botany and Plant Pathology, Oregon State University

* to whom correspondence should be addressed: [john.henning@ars.usda.gov](mailto:john.henning@ars.usda.gov), [david.hendrix@oregonstate.edu](mailto:david.hendrix@oregonstate.edu)

**Assembly Deduplication**

The first step toward identifying homology between primary contigs entailed aligning all primary contigs to each other with megablast. An example command is:

megablast -i contig.fasta -d assembly.fasta -o contig_vs_assembly.outfmt8 -e 1e-5 -m 8 -F F -W 28

Contig pairs with significant homology and low duplication frequency were tagged as candidate HPC pairs and further assessed with LASTZ and mummer. Low duplication frequency was assessed with this command:

python printLowDuplicationFrequencyBlastHits.py outfmt8.megablast.txt > lowDuplicationFrequencyBlastHits.txt

An example LASTZ command is:

lastz HPC.fasta primaryContig.fasta --gfextend --hspthresh=20000 --chain --gapped

--output=HPC_vs_primaryContig.txt --format=

general:score,name1,strand1,size1,zstart1,end1,name2,strand2,size2,zstart2,end2,identity,continuity,coverage --inner=10000 --identity=80 --strand=plus

An example mummer command is:

mummer -maxmatch -b -c -l 100 primaryContig.fa HPC.fa > primaryContig_vs_HPC.mums

To visualize alignments, mummerplot was used, and an example command is:

mummerplot -postscript -p primaryContig_vs_HPC primaryContig_vs_HPC.mums

Alignment density and coverage values were obtained from this script:

python computeNonOverlappingScoreDensity.py lastzOutputFileList.txt > results.txt

Candidate HPCs for a given primary contig were compiled into clusters with this script:

perl processLASTZ.pl results.txt primaryContigLengths.txt

Deduplication was performed with this command:

python deduplicateContigs.py clusters.txt primaryContigLengths.txt lastzOutputFileList.txt purge_haplotigs_lastzOutputFileList.txt listOfPrimaryContigs.txt listOfHaplotigs.txt curated.contig_associations.log outBaseName

**Repeat Masking**

*De novo* identification of LTR sequences in hop was performed by first creating a suffix array index for the genome assembly with this command:

gt suffixerator –db primaryAssembly.fasta –indexname hopsindex -tis -suf -lcp -des -ssp –dna

Next, analysis with LTRharvest was performed with this command:

gt ltrharvest -index hopsindex -out hops.out -outinner hops.outinner -gff3 hops.gff > harvScreen.out

Next, LTR_FINDER was run with this command:

ltr_finder -w2 primaryAssembly.fasta > findScreen.out.

Lastly, LTR_retriever was run with this command:

LTR_retriever -genome primaryAssembly.fasta -inharvest harvScreen.out -infinder findScreen.out -threads 16

Repeat masking was performed on the assembly using the library of non-redundant *de novo* hop LTRs with this command:

RepeatMasker -lib hopLTRs.fasta -pa 16 -gff contig.fasta -dir repMask_contigName

Repeat masking was also performed using a database of repeats from MIPS PlantDB using this command: RepeatMasker -lib mipsREdat_9.3p_Eudicot_TEs.fasta -q -xm -small -gff

-dir repMask_contigName/ contig.fasta

The output of running RepeatMasker on the *de novo* LTRs and MIPS PlantDB was combined and used for all downstream analyses.

**Transcriptome Assembly**

Transcriptome assemblies from leaf, meristem, and stem were generated by first creating hisat2 indexes with this command:

hisat2-build hopCascade.fasta hopCascade

RNA-seq was aligned to the assembly with hisat2 using this command, which is only shown for leaf tissue:

hisat2 --rna-strandness FR --no-discordant --no-mixed --dta -x HISAT2_INDEXES/hopCascade -1 leaf1_pair1.fastq,leaf2_pair1.fastq -2 leaf1_pair2.fastq,leaf2_pair2.fastq -S hopCascade_vs_leaf.sam

The sam file was converted to bam using this command:

samtools view hopCascade_vs_leaf.sam -bS -o hopCascade_vs_leaf.bam

Then, the bam file was sorted by coordinate using this command:

samtools sort hopCascade_vs_leaf.bam hopCascade_vs_leaf_coordSorted.bam

RNA-seq alignments were assembled into transcripts with stringtie using this command:

stringtie hopCascade_vs_leaf_coordSorted.bam -j 2 -o hopCascade_vs_leaf_coordSorted.gtf --fr -A hopCascade_vs_leaf_coordSorted.tab

All three tissue-specific RNA-seq transcriptome assemblies were merged with cuffmerge using this command:

cuffmerge -o hopCascade hopCascade.txt

The ‘hopCascade.txt’ file contains paths to each tissue-specific transcript assembly.

**Protein-Coding Transcript Prediction**

Protein coding transcripts were generated from transcript assemblies using TransDecoder. The first step was to extract transcripts from the assembly based on coordinates from the merged transcript assembly GTF file using this command:

TransDecoder-3.0.1/util/cufflinks_gtf_genome_to_cdna_fasta.pl merged.gtf hopCascade.fasta > hopCascadeTranscripts.fasta

The next step required getting the longest open reading frame (ORF) with this command:

TransDecoder-3.0.1/TransDecoder.LongOrfs -t hopCascadeTranscripts.fasta

Homology to Embryophyta UniProt genes and Pfam domains was identified with blastp and hmmscan. Alignment to Embryophyta UniProt genes was performed with this command:

blastp -query longest_orfs.pep –db embryophytaUniprot.fasta -outfmt 6 -evalue 1e-5

-num_threads 10 > hopCascade.outfmt6

Protein domain homology was assessed with this command:

hmmscan --cpu 8 --domtblout hopCascade.domtblout Pfam-A.hmm longest_orfs.pep

For TransDecoder training, the longest ORF CDS from *A. thaliana* TAIR10 was obtained with this command:

TransDecoder-3.0.1/TransDecoder.LongOrfs -t A_Thaliana.TAIR10.cds.fa

Protein-coding transcript prediction was performed using this command:

TransDecoder-3.0.1/TransDecoder.Predict -t hopCascadeTranscripts.fasta

--retain_pfam_hits hopCascade.domtblout --retain_blastp_hits hopCascade.outfmt6

--train arabidopsisLongestORFs.cds.fasta

Only the longest ORF peptides and CDS were retained.

**Hint Files for Gene Prediction**

Gene prediction hint files were created from alignments to RNA-seq, predicted protein-coding transcripts from TransDecoder, Embryophyta UniProt genes, and hop-specific ESTs. All hints were compiled into contig-specific hint files. The first step in the hint preparation pipeline was to align the RNA-seq, genes, and ESTs to the assembly. A first-pass at detecting homology was performed with blast programs, blastx and megablast. First, a blastx search was performed by aligning each contig to Embryophyta UniProt genes. An example of the blastx command is:

blastx -query contig.fasta -db embryophytaUniprot.fasta -out contig_vs_embryophytaUniprot.txt -evalue 1e-5 -outfmt 6 -max_hsps 1

Next, genes with a hit to a given contig were compiled in contig-specific fasta files and aligned to that contig with exonerate. An example exonerate command is:

exonerate --model protein2genome -q uniprotHits.fasta -t contig.fasta --showtargetgff yes --showalignment no -Q protein -T dna --forcegtag true --maxintron 100000 > contig_vs_uniprotHit.gff

Hop-specific ESTs from NCBI and TrichOME database were aligned using the same parameters. First, each contig was aligned to the ESTs with megablast. An example of this command is:

megablast -i contig.fasta -d ESTs.fasta -o contig_vs_ESTs.txt -e 1e-5 -F F -m 8 -W 28

Next, contig-specific fasta files containing EST sequences with a hit to a given contig were compiled and aligned to that contig using exonerate. An example of an exonerate command is:

exonerate --model est2genome -q estHits.fasta -t contig.fasta --showtargetgff yes

--showalignment no -Q dna -T dna --forcegtag true --percent 70 > contig_vs_estHits.gff

RNA-seq-based hint files for gene prediction were generated by first merging all tissue-specific transcriptome assemblies with this command:

samtools merge -nr mergedHopCascade.bam hopCascade_vs_leaf.bam hopCascade_vs_meristem.bam hopCascade_vs_stem.bam

The merged bam file was then sorted with this command:

samtools sort -m 5000000000 mergedHopCascade.bam mergedHopCascade_coordSorted.bam

Intron hints were generated using bam2hints with this command:

bam2hints --intronsonly --source=W --in= mergedHopCascade_coordSorted.bam

--out= mergedHopCascade_coordSorted.intron.hint

To obtain exon hints, the bam file was converted to wig format using this command:

perl bam2wig mergedHopCascade_coordSorted.bam

Bam2wig also required the indexed bam file in the same directory, which was created using this command:

samtools index mergedHopCascade_coordSorted.bam mergedHopCascade_coordSorted.bam.bai

Next, exon hints were obtained using wig2hints with this command:

cat mergedHopCascade_coordSorted.wig | perl wig2hints.pl --width=10

--margin=10 --minthresh=2 --minscore=4 --prune=0.1 --src=W

--type=exonpart --radius=4.5 --pri=4 --strand='.' > mergedHopCascade_coordSorted.exonpart.hint

Hints from TransDecoder protein-coding transcripts were obtained by first aligning the longest, complete ORF CDS to the assembly with megablast using this command:

megablast -i contig.fasta -d hopCascadeLongestCompleteORF.cds.fasta -o contig_vs_transdecoderCDS.txt -e 1e-5 -F F -m 8 -W 28

Contig-specific fasta files containing CDS with a hit to a given contig were compiled and aligned to that contig using exonerate with this command:

exonerate --model est2genome -q transdecoderHits.fasta -t contig.fasta --showtargetgff yes --showalignment no -Q dna -T dna --forcegtag true --maxintron 100000 > contig.gff

Augustus-compatible hint files were then generated.

**Gene Prediction**

Gene prediction was performed on each contig individually with Augustus. An example command is:

augustus --strand=both --genemodel=complete

--extrinsicCfgFileaugustus.2.7/config/extrinsic/extrinsic.custom.cfg

--species=arabidopsis contig.fasta --gff3=on --hintsfile=contig.hint

--outfile=contig.gff

**Error Rate Detection with GATK4**

Build index for reference assembly:

STAR --runMode genomeGenerate --runThreadN 8 --genomeDir cascadePacBioMasked --genomeFastaFiles reference.fasta

Perform first-pass alignment of RNA-seq to the assembly:

STAR --runMode alignReads --genomeDir cascadePacBioMasked --readFilesIn leaf1.fastq leaf2.fastq --outSAMmapqUnique 60 --runThreadN 10 --outFileNamePrefix leaf --outFilterMultimapNmax 1

Build index for second-pass alignment:

STAR --runMode genomeGenerate --genomeDir 2ndPassIndex --genomeFastaFiles reference.fasta --sjdbFileChrStartEnd leafSJ.out.tab --sjdbOverhang 75 --runThreadN 10

Perform second-pass alignment of RNA-seq to the assembly:

STAR --runMode alignReads --genomeDir 2ndPassIndex --readFilesIn leaf1.fastq leaf2.fastq --outSAMmapqUnique 60 --outFileNamePrefix leaf_2ndPass --runThreadN 1 --outFilterMultimapNmax 1

Extract unaligned RNA-seq reads:

gatk --java-options "-Djava.io.tmpdir=tempDir" FastqToSam -F1 leaf1.fastq -F2 leaf2.fastq -O leaf.unaligned.bam -SM leaf

Merge aligned and unaligned RNA-seq reads:

gatk --java-options "-Djava.io.tmpdir=tempDir" MergeBamAlignment -ALIGNED leaf_2ndPassAligned.out.sam -UNMAPPED leaf.unaligned.bam -O leaf_merge_alignments.bam -R reference.fasta

Add read groups to RNA-seq:

gatk --java-options "-Djava.io.tmpdir=tempDir" AddOrReplaceReadGroups -I leaf_merge_alignments.bam -O leaf_merge_alignments.rgAdded.bam -SO coordinate -RGID cascadeLeaf -RGLB lib1 -RGPL illumina -RGPU machine -RGSM leaf

Mark duplicate reads:

gatk --java-options "-Djava.io.tmpdir=tempDir" MarkDuplicates -I leaf_merge_alignments.rgAdded.bam -O leaf.dedupped.bam --CREATE_INDEX True --VALIDATION_STRINGENCY SILENT -M output.metrics

Split reads where Ns occur to obtain exon fragments:

gatk --java-options "-Djava.io.tmpdir=tempDir" SplitNCigarReads -R reference.fasta -I leaf.dedupped.bam -O split.bam

Perform first round of base recalibration:

gatk --java-options "-Djava.io.tmpdir=tempDir" BaseRecalibrator -R reference.fasta -I split.bam --known-sites hop.snps.vcf -O recal_data.table

Apply base quality score recalibration:

gatk --java-options "-Djava.io.tmpdir=tempDir" ApplyBQSR -R reference.fasta -I split.bam --bqsr-recal-file recal_data.table -O bqsr.bam

Perform second round of base recalibration:

gatk --java-options "-Djava.io.tmpdir=tempDir" BaseRecalibrator -R reference.fasta -I bqsr.bam --known-sites hop.snps.vcf -O recal_data.v2.table

Identify variants with HaplotypeCaller:

gatk --java-options "-Djava.io.tmpdir=tempDir" HaplotypeCaller -R reference.fasta -I bqsr.bam -O variants.vcf

Filter variants:

gatk --java-options -Djava.io.tmpdir=tempDir VariantFiltration -R reference.fasta -V variants.vcf -cluster-size 3 --cluster-window-size 35 --filter-name 'QD' --filter-expression 'QD < 2.0' --filter-name 'FS' --filter-expression 'FS > 30.0' -O filteredVariants.vcf

**Analysis of Coding Sequences**

Associate contigs were aligned to all primary contigs using LASTZ. An example command is:

lastz primaryContig.fasta associateContig.fasta --gfextend --hspthresh=20000 --chain

--gapped --output=primaryContig_vs_associateContig_lastzPlusStrand.txt --format=maf+

--inner=10000 --identity=80 --strand=plus

Primary contig gene model coordinates were projected to the corresponding positions in the haplotig alignment blocks, and CDS from primary contig and haplotig alignment blocks were then extracted. CDS alignments were processed with MACSE (Ranwez et al. 2011) using the exportAlignment sub-program. An example exportAlignment command is:

java -jar macse_v2.03.jar -prog exportAlignment -align primary_vs_haplotig.cds.fasta

-codonForInternalStop NNN -codonForInternalFS --- -charForRemainingFS - -out_NT primary_vs_haplotig.aligned.cds.fasta -out_AA primary_vs_haplotig.aligned.cds.fasta

**Phylogenetic Tree and Co-linearity Analysis**

A phylogenetic tree of hop and *C. sativa* was constructed by first aligning hop gene models to UniProt Embryophyta genes in both directions with these commands:

blastp –query hopGeneModels.pep.fasta –db embryophytaUniprot.fasta -evalue 1e-5

-outfmt '6 std qcovs' -out hop_vs_uniprot.txt

blastp –query embryophytaUniprot.fasta –db hopGeneModels.pep.fasta -evalue 1e-5

-outfmt '6 std qcovs' -out uniprot_vs_hop.txt

Gene models from *C. sativa* were also aligned to UniProt Embryophyta genes in both directions using these commands:

blastp –query cs10.protein_coding.2905.PEP.fasta –db embryophytaUniprot.fasta -evalue 1e-5 -outfmt '6 std qcovs' -out cannabis_vs_uniprot.txt

blastp –query embryophytaUniprot.fasta –db cs10.protein_coding.2905.PEP.fasta -evalue 1e-5 -outfmt '6 std qcovs' -out uniprot_vs_cannabis.txt

Significant hits to CBDAS and CBDAS-like, BBE-like, and THCAS and THCAS-like genes were extracted for both hop and *C. sativa* with these commands:

python getHopGeneModelSeqs.py hopGeneModels.pep.fasta hop_vs_uniprot.txt uniprot_vs_hop.txt uniprotEmbryophyta.fasta

python getCannabisGeneModelSeqs.py cannabisGeneModels.pep.fasta cannabis_vs_uniprot.txt uniprot_vs_cannabis.txt uniprotEmbryophyta.fasta

The significant hit sequences from hop and *C. sativa* were combined and aligned with clustalw2 using this command:

clustalw2 -infile=allHits.fasta -type=PROTEIN -outfile=allHits.aln > allHits.clustalw2.out

Gaps were trimmed from alignments with trimal:

trimal -in allHits.aln -gappyout > allHits_noGaps.aln

The file ‘allHits_noGaps.aln’ was converted to phylip-relaxed format. Phyml was run with this command:

phyml -i allHits_noGaps.phy -d aa -m Blosum62 -c 4 -a e

The above command produced this file: allHits_noGaps.phy_phyml_tree.txt. The tree file was visualized with ETE3.

Mutual best hits were identified by first aligning significant hits from hop and cannabis to CBDAS, CBDAS-like, THCA, THCA-like, and BBE-like genes using these commands:

blastp -query hopHits.fasta -db cannabisHits.fasta -evalue 1e-5 -outfmt '6 std qcovs'

-out hop_vs_cannabis.txt

blastp -query cannabisHits.fasta -db hopHits.fasta -evalue 1e-5 -outfmt '6 std qcovs'

-out cannabis_vs_hop.txt

Regions of co-linearity were investigated and visualized with Integrative Genomics Viewer (IGV).

Additional custom scripts and details about how they were run can be found at:

<https://github.com/padgittl/CascadeHopAssembly/>

Supplementary Figure S1. Representative mummerplots of primary and associate contig alignment blocks for increasing score density values. The score density was calculated from the total score sum for a pair of contigs divided by the length of the length of the shorter contig.

Supplementary Figure S2. Histograms of parameters used to identify and reassign primary contigs as HPCs. Density values were calculated by summing all values for the alignment and dividing by the length of the shorter contig. (a) Continuity is the percentage of the alignment that does not include gaps. (b) Coverage is the percent of the shorter sequence that is included in the alignment. (c) Percent identity is the percentage of bases in the alignment that are identical. (d) Score density is the total score sum for a pair of primary contigs divided by the length of the length of the shorter contig.

Supplementary Figure S3. Length distribution of primary contigs from the final, deduplicated primary assembly, associate assembly, Shinshuwase assembly, and Teamaker assembly.

Supplementary Figure S4. Gene and LTR density per 10 Mb (Pearson correlation 0.757, p-value 0.0).

Supplementary Figure S5. For each linkage group, a scatter plot of genetic (cM) positions and physical coordinates along the cumulative length of contigs (Mb). We expect our linkage groups to correspond to a portion of the chromosomes, but distance between neighboring contigs is currently unknown.­­

Supplementary Figure S6. Histograms of percent error per contig for three tissues, including two biological replicates for each tissue. (a) Substitutions in leaf tissue; (b) indels in leaf tissue; (c) substitutions in meristem tissue; (d) indels in meristem tissue; (e) substitutions in stem tissue; (f) indels in stem tissue.

Supplementary Figure S7. (a) Venn diagram comparing orthologs between *H. lupulus,* *C. sativa*, *A. thaliana*, and *V. vinifera*. (b) Histogram of UniProt hit counts of primary genes overlapping either haplotig or HPC regions.

Supplementary Figure S8. Gene copy number density in hop and *C. sativa* showing an enrichment of hop genes at two and four gene copies.

Supplementary Figure S9. Large-scale structural variation assessed by alignment between primary contigs and their corresponding associate contigs. (a) Alignment of primary contig 000145F and its associate contigs demonstrates that haplotig alignments tend to be shorter and have greater homology than alignments to HPCs. (b) A distribution of alignment block coverage shows that haplotig alignments are more abundant at larger coverage values than HPC alignment blocks.

Supplementary Figure S10. Heterozygosity values are from primary contigs overlapping haplotigs or HPCs and Kimura distances are from the alignment blocks of primary contigs and haplotigs or HPCs.

Supplementary Figure S11. Histogram of the CDS length difference between genes in primary contigs and associate contigs. The inset figure reveals 3-base pair periodicity.

Supplementary Figure S12. The lowest Kimura distance for the top 5% of haplotig genes was used to set the Kimura distance threshold for HPC genes. The value *n* is the number of HPC genes with a Kimura distance above the threshold set by the top 5% of haplotig genes. Probability *p* was obtained from the full set of all primary genes with a GO term, and is the probability of a GO term occurring in the set of genes in the top 5% of Kimura distances. The observed value *k* is the observed number of occurrences of a GO term in the set of HPCs with the top 5% of Kimura distances. (a) Biological Processes; (b) Cellular components; (c) Molecular function.
